# Characterizing the Human Cone Photoreceptor Mosaic via Dynamic Photopigment Densitometry

**DOI:** 10.1371/journal.pone.0144891

**Published:** 2015-12-14

**Authors:** Ramkumar Sabesan, Heidi Hofer, Austin Roorda

**Affiliations:** 1 School of Optometry, University of California, Berkeley, California, United States of America; 2 College of Optometry, University of Houston, Houston, Texas, United States of America; Dalhousie University, CANADA

## Abstract

Densitometry is a powerful tool for the biophysical assessment of the retina. Until recently, this was restricted to bulk spatial scales in living humans. The application of adaptive optics (AO) to the conventional fundus camera and scanning laser ophthalmoscope (SLO) has begun to translate these studies to cellular scales. Here, we employ an AOSLO to perform dynamic photopigment densitometry in order to characterize the optical properties and spectral types of the human cone photoreceptor mosaic. Cone-resolved estimates of optical density and photosensitivity agree well with bulk estimates, although show smaller variability than previously reported. Photopigment kinetics of individual cones derived from their selective bleaching allowed efficient mapping of cone sub-types in human retina. Estimated uncertainty in identifying a cone as long vs middle wavelength was less than 5%, and the total time taken per subject ranged from 3–9 hours. Short wavelength cones were delineated in every subject with high fidelity. The lack of a third cone-type was confirmed in a protanopic subject. In one color normal subject, cone assignments showed 91% correspondence against a previously reported cone-typing method from more than a decade ago. Combined with cone-targeted stimulation, this brings us closer in studying the visual percept arising from a specific cone type and its implication for color vision circuitry.

## Introduction

Light capture by photoreceptors is the gateway for vision. The optical properties, spatial sampling and spectral topography of the photoreceptor mosaic set the primary constraints on the fidelity of luminance and color vision. The distribution and arrangement of short wavelength cone (S-cone) photoreceptors has been studied extensively in the last three decades [1–3]. However, the similarity in the morphological and biochemical characteristics of long and middle-wavelength cones (L- and M-cones) has made their differentiation challenging. Global and indirect measures of cone activity from electroretinogram, microspectrophotometry and perception have been used to determine relative cone numerosity [4–7]. *In vitro* spatially resolved methods of their differentiation are afflicted by the very step of excising tissue, rendering the tissue no longer pristine. Specifically, removing the pigment epithelium from the intact retina adversely affects cone alignment and their waveguiding properties. Nonetheless, photopigment transmittance imaging [8] and more recently, multi-electrode electrophysiology [9] has been employed to identify the cone types in a primate retina preparation.

In living human and non-human primate retina, biophysical receptor characteristics such as waveguiding efficiency, optical density and photosensitivity have primarily been explored using densitometry, albeit on spatial scales larger than single photoreceptors [10, 11]. This is primarily because visualization of photoreceptors is impeded by the blur induced by the optics of the eye. Adaptive optics (AO) technology, when applied to imaging allows bypassing the eye’s blurred optics to resolve single cells in the living retina[12]. Using an AO-based fundus camera and cone-resolved densitometry, cone types were classified for the first time in human [13, 14] and non-human primates [15]. This effort indeed marked the only time and the only optical imaging platform that has ever been used to classify the trichromatic cone mosaic in living eyes. Only a select few AO ophthalmoscopes existed at the time of the previous studies, preventing cross-validation and repeatability assays. Since then, AO fundus cameras have largely given way to scanning laser ophthalmoscopes (SLO)[16] equipped with AO [17]. The latter represents a fundamentally different imaging modality. As opposed to a full frame single-shot image capture with a CCD in fundus cameras, AOSLOs construct an image by illuminating the retina pixel-by-pixel and collecting the scattered light from the retina in a photon detector via a confocal pinhole. Overall, this leads to high axial and lateral resolution, contrast and signal-to-noise ratio. Owing to these advantages (for complete list of advantages see [18]), AOSLOs have paved the way for studying the retina *in vivo* at an unprecedented spatial scale, ranging from studies in basic neuroscience to clinical ophthalmology [19]. In visual psychophysics, accurate photometry is challenging with a scanning confocal system. However, sophisticated, high-speed retinal tracking algorithms [20] incorporated into this modality have given targeted access to the same cells repeatedly, allowing the study of human visual perception [21] and primate physiology [22] following cone-resolved stimulation.

In this report, we sought to investigate the feasibility of employing an AOSLO for studying the bleaching kinetics of photoreceptors and as a result, develop an objective spectral classification of the human cone mosaic. Classifying the cone mosaic is the first step in unraveling the circuitry between visual perception and individual photoreceptors. Moreover, cone-resolved pigment kinetics stands to play an important role in the objective evaluation of the recent genetic and stem cell therapy approaches for cone outer segment disease and color vision deficiency.

## Materials and Methods

### AOSLO System

A SLO equipped with AO, described elsewhere [23], was refined to conduct high resolution cone-resolved densitometry. Briefly, wavelengths centered at 543 nm and 842 nm were selected from a super-continuum laser source (SuperK Extreme, NKT Photonics, Birkerød, Denmark) by using a combination of long-pass and narrow band (25nm FWHM) interference filters. Both wavelengths were relatively adjusted for the chromatic vergence difference of the eye [24] and raster scanned to generate a 1.2 deg. imaging field. Light scattered from the retina was similarly split into separate collection assemblies for each wavelength. The 842 nm wavelength was used for wavefront sensing and AO correction. The 543 nm light scattered from retina was collected in a photomultiplier tube via a confocal pinhole and rendered into a continuous video stream of the retina at 30 Hz, allowing a measure of dynamic changes in cone reflectance. The latter wavelength was chosen to optimally bleach the cone photopigment and image the cones simultaneously. L and M cone photopigments have nearly equal *and* high absorptance at this wavelength while S-cones remain relatively unaffected [25]. For this reason, L/M cone photopigment absorptance changes are recordable with high image fidelity and are separable from S cones. The light dose was chosen to reach a tradeoff between maximizing signal-to-noise ratio in image capture and minimizing an excessively fast bleach of the photopigment. High fidelity imaging was possible with a low light dose between 9 and 18 × 10^6^ Troland-seconds, (3–6 μW, 1.2 deg field of view, 4.5–9 times below the maximum permissible exposure [26]), less than half the value used previously [13]. This ensured a dense temporal sampling of absorptance kinetics and an overall larger measurable change in image intensity engendered by the photopigment.

### Subjects

The University of California Berkeley institutional review board approved this research and all subjects signed an informed consent before their participation in this study. All procedures involving human subjects were in accordance with the tenets of the Declaration of Helsinki. Four color normal male subjects and one male protanope were enrolled in the study. One of the color normal retinae was cone-typed earlier using methods described previously and his cone assignments are published in Hofer et al. (subject MD). His LMS cone assignments at 1.25 deg nasal eccentricity were compared against the methods undertaken in this study. In all other subjects measured, 1.5 deg temporal retina was imaged. The color deficiency in the protanope was verified earlier using genetic screening and his cones were classified previously, albeit at a different eccentricity compared to this study [13, 27].

### Bleaching Model

It is first important to note the relationship between residual cone photopigment and how it translates to AO cone reflectance images. The photopigment concentration density present in a cone at any time instant, *p(t)* is proportional to the incident light (*I*_*in*_) on the retina and the remaining photopigment [10]. The proportionality constant is defined by the parameter *Q*_*e*_, the amount of energy required to deplete the photopigment to *1/e* of its initial value, and was estimated to be ~3x10^6^ td-sec by Rushton & Henry. This relationship is described as following and results in an exponential decay of the form described below:

$$\frac{dp}{dt}=-\frac{I_{in}}{Q_{e}}p\left(t\right)$$

$$p\left(t\right)=P_{0}{\text{e}}^{-\left(\frac{I_{in}}{Q_{e}}\right)t}$$

Here, *P*_*0*_ denotes the concentration density at t = 0. The amount of light absorption recorded in an image (*I*_*pigment absorption*_) by a cone with photopigment density *P(t)* is scaled by its optical scattering and pigment absorption coefficients, *α*_*optical*_ and *α*_*pigment*_ respectively. This expression is described as:

$$I_{pigment\;absorption}\left(t\right)=\alpha_{optical}.\alpha_{pigment}.I_{in}.p\left(t\right)$$

Finally, the double pass light intensity exiting the cone (*I*_*double pass at cone exit*_) can be described by accounting for the light scattered by the cone independent of its photopigment concentration (*I*_*optical*_). At the limit when all the photopigment is bleached, the double pass cone image intensity is simply the incident light intensity scaled by its optical scattering coefficient.

$$\begin{array}{ccc} {\text{I}}_{\text{double}\;\text{pass}\;\text{at}\;\text{cone}\;\text{exit}}(\text{t}) & \;=\; & {\text{I}}_{\text{optical}}-{\text{I}}_{\text{pigment}\;\text{absorption}}(\text{t})\\ & \;=\; & {\text{α}}_{\text{optical}}.{\text{I}}_{\text{in}}-{\text{α}}_{\text{optical}}.{\text{α}}_{\text{pigment}}.{\text{I}}_{\text{in}}.\text{p}(\text{t})\\ & \;=\; & {\text{α}}_{\text{optical}}.{\text{I}}_{\text{in}}.[1-{\text{α}}_{\text{pigment}}.{\text{P}}_{0}{\text{e}}^{-(\frac{{\text{I}}_{\text{in}}}{{\text{Q}}_{\text{e}}})\text{t}}] \end{array}$$

### Cone-Resolved Densitometry

Subjects were first dark-adapted for 5 minutes to allow the regeneration of cone photopigments to about 90% of their maximum density. Following dark adaptation, two differing bleaching protocols were adopted for categorizing S-cones and L/M cones respectively. First, S-cones were separated by simultaneous photopigment bleaching and AO-corrected imaging at 543 nm wavelength, immediately following dark adaptation. Next, to distinguish between L & M cones, a separate partial and selective bleaching step was added between dark adaptation and imaging. This was performed to prepare the L and M cones in a state which elicits the maximum difference in their absorptance prior to bleaching and imaging with 543 nm wavelength. Two such selective bleaches were used: a 680 nm wavelength was used to selectively bleach the L cones more (~15x) than M cones, while a 470 nm wavelength was used to selectively bleach the M cones more (~1.8x) than L cones. With either wavelength, however, L *and* M photopigments are bleached simultaneously, albeit differentially, necessitating a careful selection of optimum bleaching doses. In the extreme cases, i.e. either with a very low or high light dose, L and M cones will have nearly same pigment concentrations; maximum and fully depleted respectively. The same analogy can be drawn for the case where a retina has skewed L:M ratios. Therefore, we first set the selective bleaching doses via model calculations, assuming an equal 1:1 LM cone concentration, and then titrate them empirically to maximize the difference in L and M photopigment.

### Image Processing

Five-second AO retinal videos were collected under each condition. The videos were discarded if they did not have appropriate bleach levels, had excessive eye movements or had poor image quality due to tear film breakup or inadequate optical correction. The selected retinal videos were appended together and registered offline using image stabilization software. The maximum region of interest, uncorrupted by eye motion, was selected from an averaged high signal-to-noise ratio retinal image and cone locations were identified within this region. The mean image intensity within a 0.6 arc-min square area surrounding the cone center was tracked over time and normalized.

The time course of the normalized mean intensity of cones was fit with a bleaching model of the form below, according to the relationship between cone reflectance and photopigment concentration described previously.

$$I\left(t\right)=\;\gamma -\beta e^{-\frac{t}{\tau }}$$

The parameters *γ*, β and τ were obtained from a non-linear least squares fit. The difference between the final and initial intensity computed from the fitted parameters gave a relative measure of pigment absorptance under each bleach condition.

### Clustering Analysis

**Fig 1:**
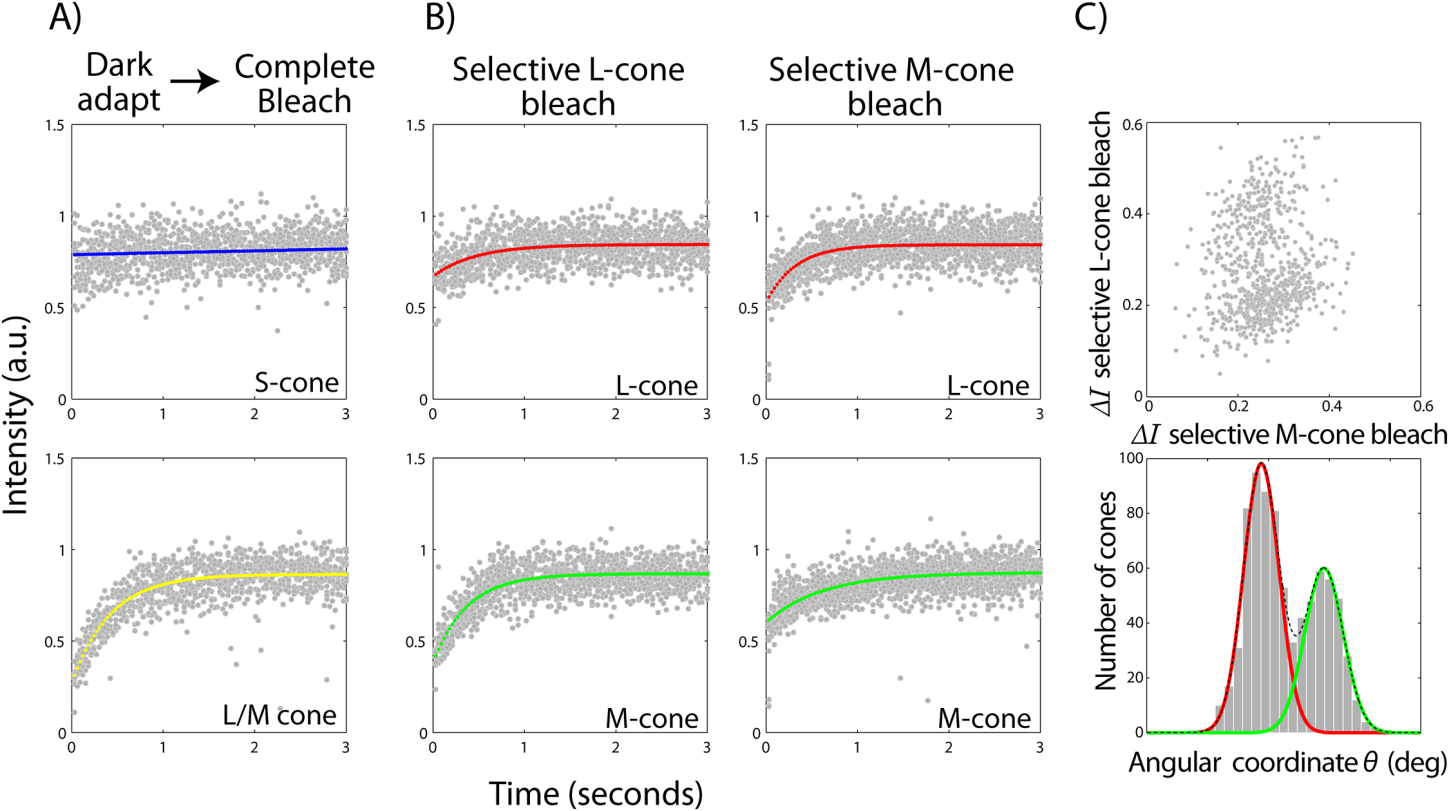
Methodology for classifying LMS cones. Representative examples of cone bleaching curves under a complete bleach from a dark adapted retina (A) and under a selective L and M-cone (B) bleach respectively are shown. Mean intensity timecourses for a given cone are accumulated together across all bleaching cycles. Non-linear least squares fit to the mean intensity of individual cones are shown and colored according to their peak sensitivity as blue, green or red for LMS cones respectively. The change in intensity under selective L- and M-cone bleaches for all L and M cones are plotted in (C) in a scatter plot. Each scatter point represents either an L or M cone. The x-y plot is converted to polar coordinates. The histogram of the angular coordinate is separared into respective L and M cone clusters by fitting a sum of 2 one-dimensional gaussian curves and taking the point of intersection of the component gaussians.

For classifying S-cones, a histogram of the fractional change in cone image intensities obtained by going from a dark adapted to a completely bleached retina was fit with a sum of two 1-dimensional Gaussians. Each Gaussian represented S and L/M cones respectively. For delineating L and M cones, the change in cone image intensities under selective L-cone and M-cone bleaching were plotted in an x-y plot and converted to polar coordinates (Fig 1). A histogram of the angular polar coordinate was fit similarly with a sum of two 1-dimensional Gaussians. The clustering method followed here is analogous to a 2-component, 1-dimensional Gaussian mixture model [28]. The intersection of the component Gaussians separated the clusters. The probability of any cone belonging to a particular cluster, S or L/M for the former and L or M for the latter, was obtained from the ratio of the sum Gaussian to its individual components.

## Results

### Bleaching Kinetics

Fig 1A & 1B show representative examples of cone bleaching curves under a complete bleach from a dark adapted retina and under a selective L and M-cone bleach respectively. The mean intensity of the entire image nearly doubles upon a complete bleach following dark adaptation, indicative of the amount of photopigment signal available for selective densitometry.

Optical parameters obtained from the cone-resolved bleaching model are shown in Table 1. First, the estimate of double-pass optical density of individual cones was obtained. It is defined as the product *α*_*optical*_ *α*_*pigment*_ in the dark adapted to complete bleach condition. This measure does not however account for the effects of stray light, and hence is an underestimate of the actual double-pass density, a limitation similar to previous studies. Second, the photosensitivity (1/Qe) of individual cones was measured. At the level of individual cones, this parameter depends on their optical density and photopigment sensitivity at the imaging wavelength. The remaining constants encompassed in photosensitivity are related to the probability of photoisomerization in cones. Both double pass optical density and photosensitivity agree well with previous estimates [10, 29]. Overall, these parameters are found to be similar across both L and M cones in each subject. The mean absorptance in cones (removing S-cones, explained later) obtained from the dark adapted to complete bleaching paradigm is finally also indicative of the variation in apparent optical density, and of the available photopigment in L and M cones (Table 1).

**Table 1:** Cone optical properties and classification of cone-subtypes.

Subject/Age/Ethnicity	Double pass optical density*(log10 units)		Photosensitivity*(1/Q_e_)x 10^−7^ td^-1^.sec^-1^		normalized absorptance*		%S cones	L:M ratio	% L/Merror
	L cones	M cones	L cones	M cones	L cones	M cones			
S1 /32 /C	0.28 ± 0.09	0.27 ± 0.08	1.69 ± 0.50	1.70 ± 0.49	0.52 ± 0.12	0.53 ± 0.11	5.26	1.96:1	3.23
S2 /37 /C	0.30 ± 0.09	0.30 ± 0.09	1.80 ± 0.58	1.86 ± 0.63	0.50 ± 0.12	0.50 ± 0.11	4.90	2.48:1	4.37
S3 /32 /A	0.36 ± 0.07	0.36 ± 0.08	1.92 ± 0.37	2.03 ± 0.45	0.43 ± 0.08	0.43 ± 0.08	4.94	1.92:1	3.44
S4 /39 /H	0.41 ± 0.11	0.39 ± 0.11	2.05 ± 0.52	2.07 ± 0.52	0.39 ± 0.11	0.41 ± 0.12	4.92	1.48:1	4.17
P1 /44 /C	--	0.28 ± 0.08	--	2.03 ± 0.74	--	0.53 ± 0.11	6.40	protanope	

*Mean ± standard deviation across all analyzed cones except S-cones at 543 nm wavelength.

C: Caucasian, A: Asian, H: Hispanic

### Separating *LMS* Cones

**Fig 2:**
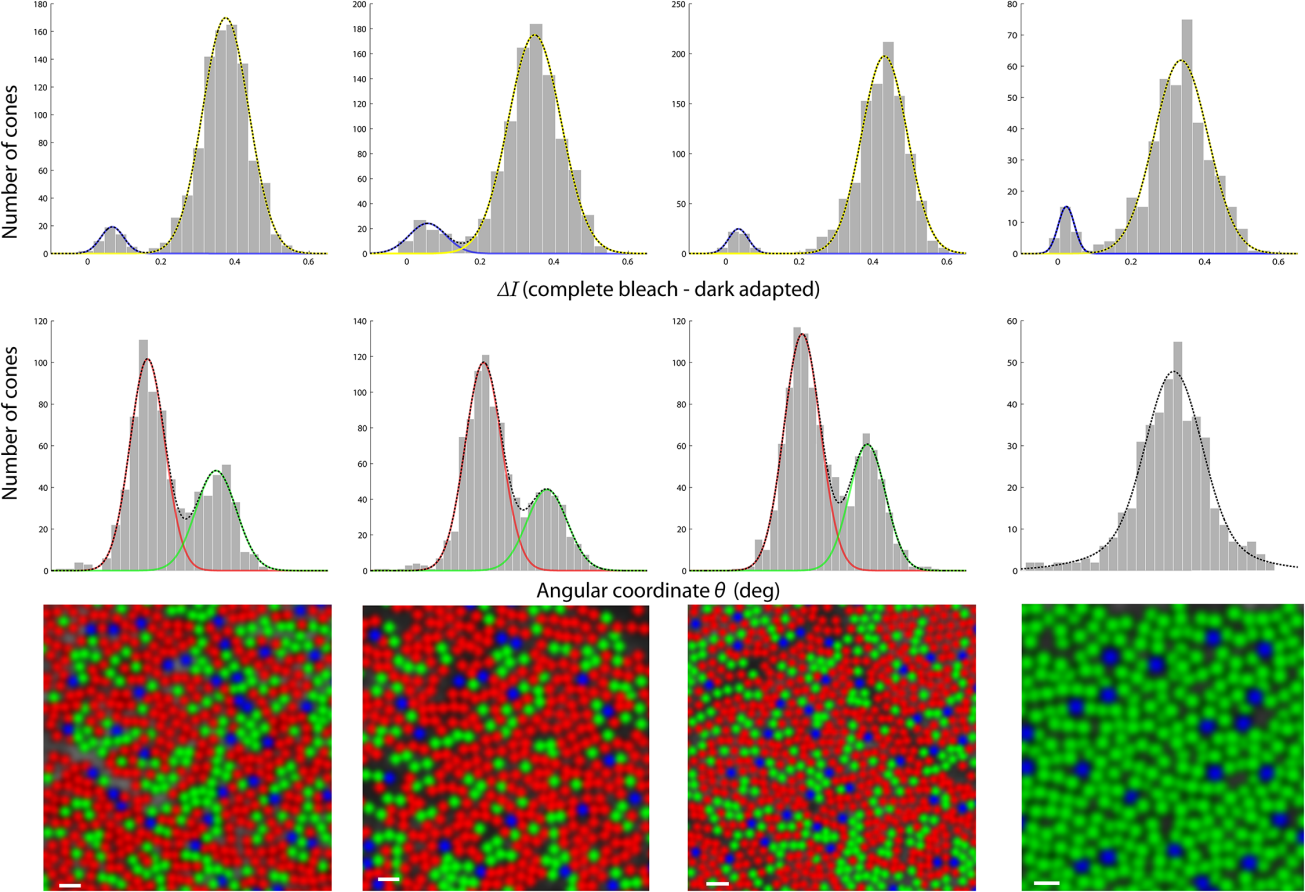
LMS cone mosaics in the retinae of human subjects. Each column represents an individual subject’s retina at 1.5 deg temporal eccentricity. The last column represents cone assignments for the protanopic subject. S-cones appear as a cluster of weakly reflecting cones under complete bleach starting from a dark adapted retina (top row). L and M cones appear as two distinct clusters of cones on the basis of their relative change in intensity under selective bleaches (middle row). No separation of such clusters are obtained in the protanope. Based on where a cone appears in the S vs L/M and L vs M clustering analysis, it is shaded as ‘blue’, ‘green’ and ‘red’ to represent S, M and L cones respectively (bottom row). Other than S-cones, cone assignments for the protanope are shaded as ‘green’ M-cones on the basis of previous verification of his color deficiency. The scale bar is 2 arc-min.

Fig 1A shows how a candidate S-cone is separated from L/M cones on the basis of their intensity change under 543nm bleaching and imaging following dark adaptation. Given that the S-cone opsin remains relatively unaffected by this wavelength, it is expected to exhibit negligible changes in its absorption characteristics. As a result, negligible changes in image intensity will be observed in cone reflectance. The converse is expected for L and M cones under this bleaching protocol, i.e. their photopigments will be bleached progressively leading to decay in their absorptance and increase in their image intensity. Cone-resolved change in intensity so obtained is separated into S and L/M categories on the basis of their magnitude (Fig 2A).

Fig 1C shows a representative scatter plot of change in cone intensities under selective L and M-cone bleach. Transformation to polar coordinates yields an estimate of variance in optical density (radial coordinate) and the relative impact of the selective bleaches on cone intensities (angular coordinate). The latter is separated into corresponding clusters of L and M cones by the intersection of the two component Gaussians, obtained from the sum Gaussian fit. Fig 2B shows such cluster separations in all subjects and Fig 2C shows the corresponding LMS-cone mosaics labeled in false-color according to their peak sensitivities as ‘red, ‘green’ and ‘blue’ respectively. In these images, dark spaces correspond to shadows cast on cones by the overlying blood vessels, a feature that is accentuated by the confocality of the scanning instrument. Table 1 shows the percentage of different cone classes, the estimate of error in their assignments and the average estimated probability of correct cone classification in the 5 subjects. Overall, the error is under 5% and the probability is greater than 0.95 for identifying cone types. It is a matter of chance that all the 4 color normal subjects, 2 of whom were not Caucasian, have nearly same L:M ratios, albeit close to the population norm [5]. The cone mosaic of a protanope is also shown in Fig 2.

### Comparing AO Fundus Camera and SLO for Cone Classing

**Fig 3:**
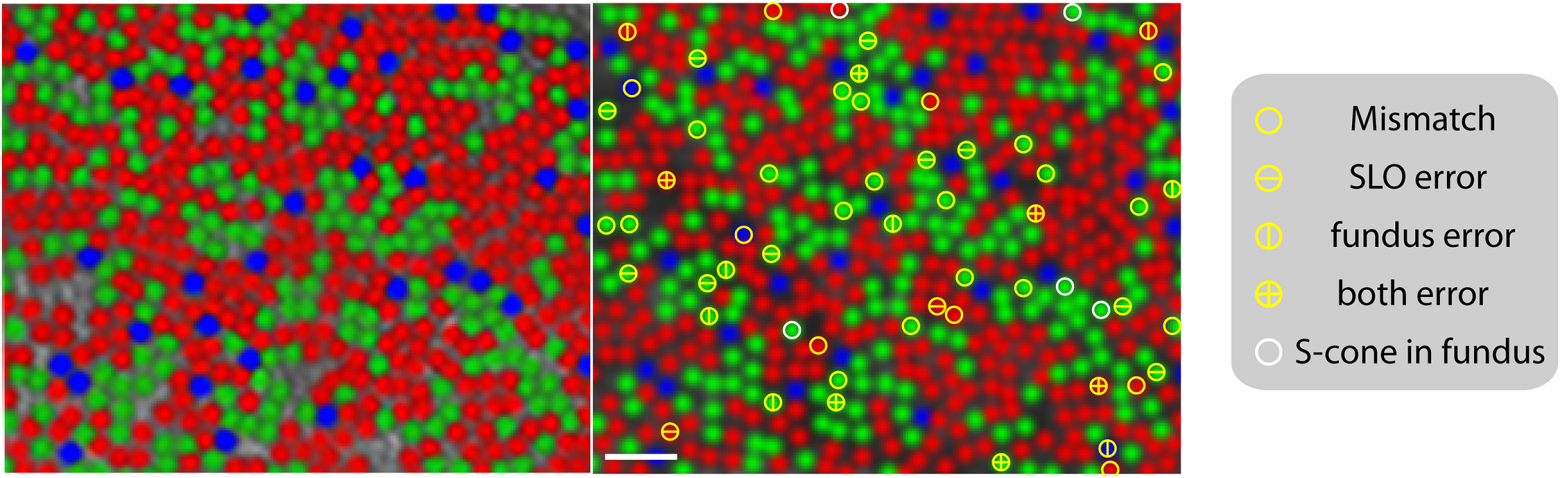
Comparison of cone classification in the AO fundus camera (left) and SLO (right). Cone assignments in the former are obtained from Hofer et al. 2005. Mismatched cones in the SLO image are marked as ‘circles’. Cones which have probability less than 0.9 to be reliably identified in the SLO and fundus are marked with ‘horizontal’ & ‘vertical’ lines respectively. The scale bar is 4 arc-min.

Given that only one optical system has ever been used to map the trichromatic mosaic *in vivo*, a comparison of the previous method with the present one is invaluable. We recruited a color normal subject whose retina had previously been classified using the AO fundus camera more than 10 years ago (subject MD in Hofer et al. 2005). Fig 3 shows the LMS mosaic of subject S4, classified in the AO fundus camera and in the AOSLO. Cone locations remained stable and aligned well with the AO fundus camera image taken previously. Total L-M classification error was ~4% in the AO fundus camera and SLO in this subject. Overall, the cone assignments obtained from the two methods have ~ 91% agreement over 650 analyzed cones. The mismatches are marked over the cones in the AOSLO image. Cones which had low probability (<0.9) to be reliably identified in the SLO and fundus camera are one source of mismatch. This low probability categorization accounts for 3.8% of the 650 analyzed cones: 1.2%, 1.8% and 0.8% in fundus camera, SLO and both modalities respectively. Second, 0.8% of cones were identified as S-cones in the AO fundus camera while they were identified as L or M cones in the AOSLO. Their individual bleaching curves were investigated for confirmation. This discrepancy arises due to the better resolution and contrast in the AOSLO which allows a more sensitive measure of photopigment density. As a result, a low pigment L/M cone is more likely to be differentiated from an S-cone. 4.2% of the cones were unaccounted within the above two criterion. Reflectivity of cones is highly variable with respect to one another. A given cone’s reflectivity can also fluctuate substantially with time, given visible stimulation, outer segment disc shedding and renewal [30–32]. Given that the site of reflectance changes is largely in the outer segment, it can lead to discrepancies in measures of photopigment density and cause independent sources of error between the two cone typing methods. Assuming that 30% of cones on average [30] exhibit a variance in their mean reflectance of as low as 0.01, error in L-M classification was estimated to be up to 5%. Taken together, the aforementioned factors establish comparable reliability between the two methods, despite the time point separation.

## Discussion

The trichromatic cone mosaic was characterized using photopigment densitometry. High-resolution AO imaging allowed obtaining photopigment-specific bleaching signatures from individual cones with high efficiency. Following bleaching, retinal reflectance is not entirely attributed to photopigment kinetics. Other factors such as interference artifacts from cone outer segment tips, [30, 31, 33, 34]light-evoked intrinsic signals [32], light scattered from inner retina and the anterior ocular optics all contaminate the reflectance signal attributable to photopigment. Therefore, care should be taken in interpreting values of density reported here or elsewhere as *true* optical density; rather they are *apparent* cone optical densities encompassing these extraneous factors. Several steps were taken in this study to mitigate such factors in determining density. Firstly, imaging was undertaken at 543nm, a wavelength for which both L and M cone photopigments have near maximum absorptance. Second, AO and confocality of the imaging instrument provided axial and lateral localization of the reflectance signals, most likely from the cone inner and outer segment junction. This ought to minimize the contribution of stray light to measures of optical density, namely light-induced scattering from other retinal layers and anterior optics. Lastly, the light dose used for bleaching and imaging was low, i.e. 3–6 μW at the cornea allowing the measure of high apparent cone optical density and dense sampling of pigment kinetics following dark adaptation. Together, these allowed dynamic bleaching kinetics to be measured from individual cones and yielded their double pass optical density, photosensitivity and absorptance. Cone-resolved bleaching kinetics has been studied previously with an AO fundus camera, yielding large variability in foveal cone optical properties [35]. Here, we measured significantly smaller variability across bleaching parameters on an individual cone-by-cone basis. Previously, most studies differentiating L and M cone optical densities were based on measures from protanopes and deutranopes. Smith & Pokorny[36] reported higher photopigment density in L-cones than M-cones using psychophysical measures. On the other hand, Renner et al.[37] reported similar L and M-cone optical densities across a large number of dichromats. Our study is in line with the latter, and with retinal densitometry performed by Berendschot et al.[38] The key differences in our measures are that they are based on trichromatic observers and represent cone-resolved measures of optical density, unlike larger stimuli used in psychophysics or densitometry previously. Overall, our measures of optical density from densitometry are lower than the single pass psychophysical estimates of Renner et al.[37], although they match with those from Rushton & Henry[10], Elsner et al.[11], and van Norren & van de Kraats.[39] Previous psychophysical estimates were found to be qualitatively similar as a function of eccentricity, but usually greater than densitometric measures of optical density[11]. Given the recent developments in cone-targeted stimulation, a spatially localized comparison of psychophysical and densitomeric measures of optical density can be envisioned. It remains to be seen whether the inter- and intra- individual variability in cone optical density correlates directly with individual cone function and continues to be an active area of investigation[40].

Measuring bleaching kinetics reliably facilitated an efficient method of classifying cone subtypes in living human retinae. The methods previously developed using the AO fundus camera by Roorda & Williams averaged as many as 50 images across 5 days for each bleaching condition while Hofer et al. used between 7 and 30 images over the course of 1–3 days. In contrast, 8 to 15 bleaching cycles per condition were needed to map the cone types here. The time taken ranged from 3–9 hours with the main limitations posed by subject fatigue and eyes’ tear film suitability for imaging. Several performance measures denoted efficacy. First, in every case, the S-cones were delineated via a minimal change in their absorptance at 543 nm wavelength bleach following dark adaptation. Second, the percent mis-assignments in L and M cones was under 5% and they are identified with probability greater than 0.95. Finally, the cone classing method reported here was verified against the only previously published method by employing the same subject and retinal patch classified more than 10 years ago. Good agreement was established between the two methods. Mismatches between the cone assignments were explained largely by independent sources of error most likely due to cone reflectivity variation. Because AO ophthalmoscopes were rare and the prior methods were time-consuming, cone assignments were not cross-validated on a different imaging platform or repeated on the same subject. This marked an important step here in the verification of either method, SLO or fundus camera.

Certain limitations remain which are albeit within reach to be overcome. First, this study and others have been unable to confirm foveal tritanopia, an S-cone free zone in the foveola [2, 3, 41]. In our case, this is primarily because of insufficient lateral resolution to resolve foveal cones, although with recent improvements in optical design of imaging systems, reliable visualization of the foveal cone mosaic is possible [42]. Second, error rates of cone mis-assignments can be improved further using deconvolution algorithms [43], which would further drive down requisite bleaching cycles and improve efficiency. The demands on these algorithms would however be different than that described by Christou et al.[43], because on a frame-by-frame basis, photon noise and low light efficiency will have a larger impact on image fidelity than optical blur. Additionally, implementing such an image deconvolution will further aid in drawing statistics on spatial arrangement of cones, i.e. whether cones of like type appear randomly or clump together. Previous studies have shown that M and L cones appear randomly, with only a small tendency towards clumping [14, 15]. Both these reports note the negative impact of blurring on clumping analyses. Moving forward, an alternative and perhaps more efficient strategy for classifying the cone-types can be contemplated by accounting for cone reflectivity variation. For instance, instead of bleaching and imaging the retina at 543nm alone, simultaneous imaging with an L-cone or M-cone selective wavelength close to their peak absorptance will allow temporally-synced normalization of reflectivity. If these changes are mainly attributable to the outer segment and hence pigment density, significant reduction in classification uncertainty is expected. Additionally, the time constant of pigment decay will be differentially affected for L and M cones with a wavelength close to their peak absorptance.

In summary, this study marks the first time that a SLO equipped with AO was used to map the cone types in human retinae using dynamic photopigment densitometry. With cone-targeted visual stimulation now becoming readily accessible in the same imaging platform [21], it is now possible to relate the specific cone type with its associated percept, color, threshold sensitivity and so on, thus setting the stage for studying perceptual correlates of spatial and color vision retinal circuitry. Moreover, with recent advances in gene therapy for color vision deficiency [44] and retinal degenerations [45], objective measures of cone opsin expression, cone function and long-term viability is likely play a pivotal role in determining outcome measures in human patients.
